# How to Behave at the Table

**Published:** 1882-03

**Authors:** 


### HOW TO BEHAVE AT THE TABLE.

Perhaps one reason why boys and girls do not feel so comfortable and so at ease as they might on special occasions at the table, is because they do not take pains to be perfectly polite when there is no one present but the ordinary home folks. In the first place, we owe it to ourselves always to look very neat and nice at our own tables. Nobody should presume to sit down to a meal without making a proper toilet beforehand. Boys ought to be careful that their hair is brushed, their hands and faces clean, their nails free from stain and soil, their collars and ties in order before they approach the table. A very few moments spent in this preparation will freshen them up and give the outward appearance of little gentlemen. I hope girls do not need to be cautioned thus.

Then there are some things which good manners render necessary, but about which every one is not informed. Of course you know that you are not to eat with your knife. Fifty years ago people frequently ate with their knives, and it is possible that now and then you may see some old-fashioned person doing so ; but it is not customary now, nor is it safe or convenient. When you send your plate for a second helping, or when it is about to be removed, you should leave your knife and fork side by side upon it.

It is not polite to help yourself too generously to butter. Salt should be placed on the edge of the plate, never on the tablecloth. Do not drink with a spoon in the cup, and never drain the very last drop. Bread should be buttered on the plate, and broken a bit at a time and eaten in that way. Eating should go on quietly and not hastily. Nothing is worse than to make a noise with the mouth while eating, and to swallow food with noticeable gulps.

Do not think about yourself, and fancy that you are the object of attraction to your neighbors.

